# Latent Trajectories and Risk Factors of Prenatal Stress, Anxiety, and Depression in Southwestern China—A Longitudinal Study

**DOI:** 10.3390/ijerph20053818

**Published:** 2023-02-21

**Authors:** Yuwen Gao, Xian Tang, Ruibin Deng, Jiaxiu Liu, Xiaoni Zhong

**Affiliations:** 1 Department of Medical Record Management, West China Second University Hospital, Sichuan University, Chengdu 610041, China; 2 Key Laboratory of Birth Defects and Related Diseases of Women and Children (Sichuan University), Ministry of Education, Chengdu 610041, China; 3 School of Public Health, Chongqing Medical University, Chongqing 400016, China; 4 Research Center for Medicine and Social Development, Chongqing Medical University, Chongqing 400016, China; 5 Chongqing Shapingba District Center for Disease Control and Prevention, Chongqing 400030, China; 6 College of Medical Informatics, Chongqing Medical University, Chongqing 400016, China

**Keywords:** prenatal stress, prenatal anxiety, prenatal depression, longitudinal trajectories, growth mixture model

## Abstract

(1) Background: Few studies have explored the heterogeneity of trajectories of stress, anxiety, and depressive symptoms during pregnancy. This study aimed to explore the trajectory groups of stress, anxiety, and depressive symptoms in women during pregnancy and the risk factors associated with those groups. (2) Methods: Data came from pregnant women recruited from January to September 2018 in four hospitals in Chongqing Province, China. A structured questionnaire was given to pregnant women, which collected basic information, including personal, family, and social information. The growth mixture model was applied to identify potential trajectory groups, and multinomial logistic regression was applied to analyze factors of trajectory groups. (3) Results: We identified three stress trajectory groups, three anxiety trajectory groups, and four depression trajectory groups. Less developed regions, inadequate family care, and inadequate social support were associated with a high risk of stress; residence, use of potentially teratogenic drugs, owning pets, family care, and social support were strongly associated with the anxiety trajectory group; family care and social support were the most critical factors for the depression trajectory group. (4) Conclusions: The trajectories of prenatal stress, anxiety, and depressive symptoms are dynamic and heterogeneous. This study may provide some critical insights into the characteristics of women in the high-risk trajectory groups for early intervention to mitigate worsening symptoms.

## 1. Introduction

Pregnancy is the most psychologically vulnerable period for women. During pregnancy, women are prone to varying degrees of stress due to changes in hormone levels and numerous other factors [1], such as brief appearances of fear, nervousness, and the possible long-term persistence of negative emotions. Negative emotions in women, such as stress, anxiety, and depression, are prevalent during pregnancy. In China, incidences of prenatal anxiety and depression symptoms range from 3.7% to 23.2% and 10.3% to 54.8%, respectively [2,3,4,5,6], while that of prenatal stress symptoms ranges from 78.9% to 92.9% and the symptoms are generally at a mild to moderate level [6,7,8,9]. In other countries, anxiety, depression, and stress symptoms were found in approximately 14.0–54.0%, 4.0–37.1% [10,11,12,13,14,15], and 22.4–84.0% of pregnant women [16,17,18].

Negative emotions during the prenatal period may pose potential risks to maternal and newborn health. Prenatal stress, anxiety, and depression are not only associated with fetal development and adverse pregnancy outcomes, such as low birth weight, preterm birth, and fetal distress [16,19,20,21,22,23], but also have more lasting effects, directly or indirectly, on the growth and development of children. Children of women who experience one or more negative emotions during pregnancy are more likely to have behavioral and emotional problems [24], attention deficit hyperactivity disorder [25], and autism in childhood [26]. They are also more likely to have depression, impulsivity, and cognitive disorders in adolescence [25,27] and to suffer from schizophrenia in adulthood [28]. In addition, women with prenatal symptoms of anxiety, depression, perceived stress, and post-traumatic stress disorder may experience more intense pain and have a higher risk of developing pregnancy complications, such as diabetes and pre-eclampsia, during childbirth [29], and they are more likely to suffer from postpartum depression during the postpartum period [30,31]. Against the background of the “two-child policy”, maternal and newborn health is crucial for a country where there are a great number of women of childbearing age and great numbers of newborns are born annually. Therefore, it is essential to study the prenatal mental health of Chinese women.

Limited research suggests heterogeneity in the duration, severity, and trajectories of stress, anxiety, and depressive symptoms during pregnancy, which may be associated with women’s sociodemographic characteristics, mental health medical history, and social/family support [10,11,13,32,33,34]. Understanding this variation is critical to developing prevention and intervention programs that target specific characteristics of prenatal stress, anxiety, or depressive symptoms. There is a paucity of studies identifying potential trajectory groups for stress, anxiety, or depressive symptoms during pregnancy in China, as we found only one trajectory study of perinatal depressive symptoms in women from Hunan Province, China [35], and there are still gaps in trajectory studies regarding stress and anxiety.

Therefore, the purpose of this study was to explore the trajectory groups of prenatal stress, anxiety, and depression and then to determine the risk factors for these trajectory groups.

## 2. Methods

### 2.1. Procedure

The data of this study were collected from the Study on the Public Opinion Propagation Model for Generative Mechanism and Regularity of Cesarean Delivery Behavior (Project No. 71,573,027), a longitudinal follow-up survey funded by the National Natural Science Foundation of China and approved by the Ethics Committee of Chongqing Medical University.

This study was carried out in Chongqing, a provincial capital city in southwestern China. Participants were enrolled from the obstetrics and gynecology departments of four hospitals in four economically different regions (developed areas: Yubei and Jiangjin districts; less developed areas: Dianjiang district and Yunyang district). Women who attended their first pregnancy screening at these four hospitals between January 2018 and September 2018 and met the following criteria were included: (a) gestational age < 15 weeks; (b) singleton pregnancy; (c) signed informed consent and expressed willingness to participate in a longitudinal study; (d) no previous history of cesarean section; and (e) no health problems (e.g., mental illness). Informed consent was obtained from all participants before study enrollment.

### 2.2. Study Content and Measurements

A structured questionnaire was used to obtain data on individual, family, and social factors for this study. The variables in the questionnaire were reviewed by public health specialists, psychologists, obstetricians, and gynecologists. A follow-up survey was conducted at early pregnancy (<15 weeks), middle pregnancy (15–27 weeks and 6 days), and late pregnancy (28 weeks–before delivery).

The content of the investigation included sociodemographic data (including age, residence, education level, employment status, and monthly per capita household income) and prenatal examination information on personal behaviors and psychological condition (stress, anxiety, and depression). Sociodemographic data were collected in the follow-up at early pregnancy. Obstetric characteristics and suspected adverse factors (such as parity, vaginal bleeding, drug use, exposure to high concentrations of industrial chemicals, owning pets, exposure to newly renovated houses, alcohol consumption, smoking, and past medical history) were obtained from the maternal and child health manuals. Some of the variables (prenatal exercise, antenatal classes, psychological condition, family care, and social support) were evaluated multiple times in the three follow-ups from early to late pregnancy. Some of these variables were redefined before the analysis: (1) prenatal exercise was defined according to the number of times pregnant women answered “yes” in the three follow-ups, ≥2—“regular” and ≤1—“never or occasionally”; (2) attendance at antenatal classes was defined according to whether the pregnant women attended antenatal classes in the three follow-ups, ≥1—“yes” and no attendance—“no”; (3) for family care and social support, cluster analysis was used to distinguish groups with different score levels in the three periods according to the corresponding scale scores. The group with high overall scores was defined as “high” and that with low overall scores as “low”.

#### 2.2.1. Stress, Anxiety, and Depression Measurements

The Pregnancy Pressure Scale (PPS), developed by Zhanghui Chen et al. [36], was used to measure stress. The PPS has 30 entries and stress is measured using the mean score calculated from the total score for all questions, with a score of 0 indicating no stress, 0.01–1.00 indicating mild stress, 1.01–2.00 indicating moderate stress, and 2.01–3.00 indicating severe stress. In this study, the Cronbach’s α coefficients for the use of the PPS in the first, second, and third trimesters were 0.936, 0.943, and 0.946, respectively.

Anxiety was measured with the Hamilton Anxiety Scale (HAMA) [36]. Fourteen items from the HAMA were used and a total score of ≤7 calculated for all items was used to indicate no anxiety, 8–14 to indicate suspected anxiety, 15–21 to indicate mild anxiety, 22–29 to indicate moderate anxiety, and greater than or equal to 30 to indicate severe anxiety. The Cronbach’s α coefficients for the HAMA in this study were 0.879, 0.914, and 0.919, respectively.

Depression was measured with the Self-rating Depression Scale (SDS) [37], which is used to calculate an index of depression severity (actual total score/maximum possible score on all items). An index below 0.5 indicates no depression, 0.50–0.59 indicates mild depression, 0.60–0.69 indicates moderate depression, and greater than or equal to 0.7 indicates major depression. In this study, the Cronbach’s α coefficients for the SDS were 0.775, 0.817, and 0.821, respectively.

#### 2.2.2. Family and Social Factors

The Family Adaptation Partnership Growth Affection (APGAR) scale uses the total score for its entries to assess maternal satisfaction with family functions [38]. A total score of 0–3 indicates low family care, 4–6 indicates moderate family care, and 7–10 indicates good family care. The Cronbach’s α coefficients for APGAR scale in this study were 0.826, 0.833, and 0.836, respectively.

The Social Support Rating Scale (SSRS), compiled by Xiao Shuiyuan in 1986, was used to measure social support [39]. A total score was calculated for all items, with scores below 35 being low, 35–45 being moderate, and above 45 being high. The Cronbach’s alpha coefficients for the SSRS in this study were 0.562, 0.625, and 0.656, respectively.

### 2.3. Statistical Analysis

Data were double-entered and validated using Epidata 3.1 software, and all statistical analyses were performed using SAS 9.4 software except for the growth mixture model (GMM), which was run with Mplus 7.4 software.

The statistical analysis was conducted in three steps. First, trajectory groups for stress, anxiety, and depressive symptoms were explored using the GMM [40], a classification model that deals with longitudinal data and assumes that individuals in a population differ in their longitudinal developmental trajectories. It clusters individuals with the same or similar developmental trajectories into one group and, ultimately, into a limited number of mutually exclusive trajectory groups, with detailed descriptions of the specific parameters of the trajectory groups [41]. We fit the model with one to four classes and set the model selection criteria. The first set of criteria included the Akaike information criterion (AIC), Bayesian information criterion (BIC), and sample size-adjusted BIC (aBIC). The smaller the value was, the better the model fit. The second criterion for classification accuracy was entropy; the larger the entropy was, the better the potential classification quality. Finally, we also considered the model fit test results (vuong-lomcndellrubin likelihood ratio test (VLRT)). *p* < 0.05 for the significant likelihood ratio test for class k indicated that the specified class k model was an improvement over the model with class k − 1 [42]. Second, descriptive statistics were used to analyze the frequencies and percentages of population characteristics. Third, one-way χ^2^ tests and multinomial logistic regression were used to analyze the risk factors associated with each trajectory, setting the trajectory groups of stress, anxiety, and depressive symptoms as outcome variables. Two-tailed *p* < 0.05 was the significance level for the logistic regression. Demographic and psychological factors collected by the survey were used in the one-way χ^2^ tests and multinomial logistic regression.

## 3. Results

### 3.1. Subject Characteristics

A total of 916 women (developed regions: 355, less-developed regions: 561) from four hospitals were recruited for this study and they underwent three surveys from early to late pregnancy. The age of the participants ranged from 16 to 44 years, and 67.5% were primiparous. Of these, 62.2% lived in urban areas and 37.8% in rural areas. A total of 39.4% of the women had completed higher education, and 41.5% of them had a per capita monthly income between CNY 3000 and 5000. Women with per capita monthly household income above CNY 5000 accounted for 33.1%, while those with income below CNY 3000 accounted for 25.3%. More than half (50.8%) of the women were housewives or jobless.

### 3.2. Prevalence of Perinatal Stress, Anxiety, and Depression

Based on the psychological scale scores of the 916 women in the three periods and the division of the scores (See Section 2), we explored the horizontal prevalence of stress, anxiety, and depressive symptoms in early, middle, and late pregnancy among these women (see Table 1). The prevalence of prenatal stress was high and symptoms were mild (76.75%, 78.28%, 78.06%), with only 7.75% of women being free of stress symptoms in early pregnancy. The prevalence levels of anxiety and depression were lower than that of stress symptoms, with the absence of anxiety symptoms remaining consistently above 85% and the absence of depressive symptoms consistently above 95%.

### 3.3. Identifying Trajectory Groups of Prenatal Stress, Anxiety, and Depressive Symptoms

Based on the minimum principle defined by the AIC, BIC, and aBIC and the maximum principle defined by the entropy, as well as the VLRT test (*p* < 0.05),we identified three stress trajectory groups (BIC = 2104.410; aBIC = 2059.947; entropy = 0.892), three anxiety trajectory groups (BIC = 16,822.106; aBIC = 16,777.644; entropy = 0.903), and four depression trajectory groups (BIC = −7708.881; aBIC = −7769.223; entropy = 0.934) as the best fitting models (see Appendix A). The means and confidence intervals for the trajectory subgroups are shown in Table 2, and the Bonferroni method was used for multiple comparisons of different trajectory subgroups at the same follow-up time point. The multiple comparisons were statistically significant for the stress, anxiety, and depression subgroups at all three follow-up time points, which may indicate that the trajectory analysis model grouping was effective. The distributions of symptom levels for trajectory subgroups at each follow-up point are shown in Table 3.

#### 3.3.1. Trajectory Groups of Prenatal Stress Symptoms

**Figure 1:**
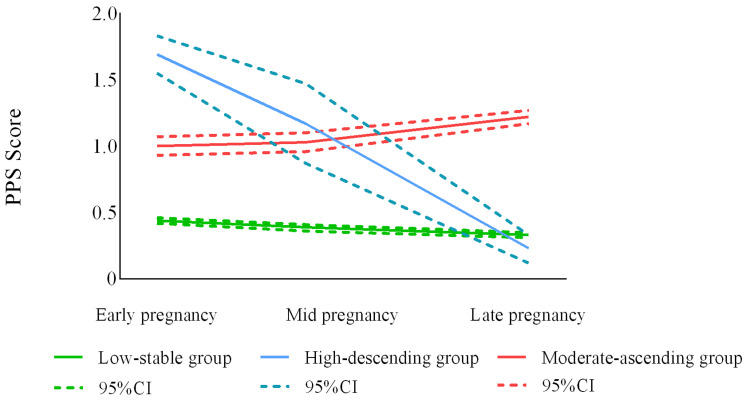
Optimal model trajectory curves for prenatal stress (n = 916).

Among the three stress trajectory groups (Figure 1), the largest group was the “low-stable” (n = 750, 81.9%) group, with women in this group showing low scores at the three follow-up time points and reporting no or mild stress symptoms. The second largest was the ”moderate-ascending” group (n = 142, 15.5%), with some women in this group reporting moderate or severe stress symptoms that became worse with time. Women in the “high-descending” group (n = 24, 2.6%) reported the highest baseline levels but, over time, their “early pregnancy stress” symptoms gradually decreased.

#### 3.3.2. Trajectory Groups of Prenatal Anxiety Symptoms

**Figure 2:**
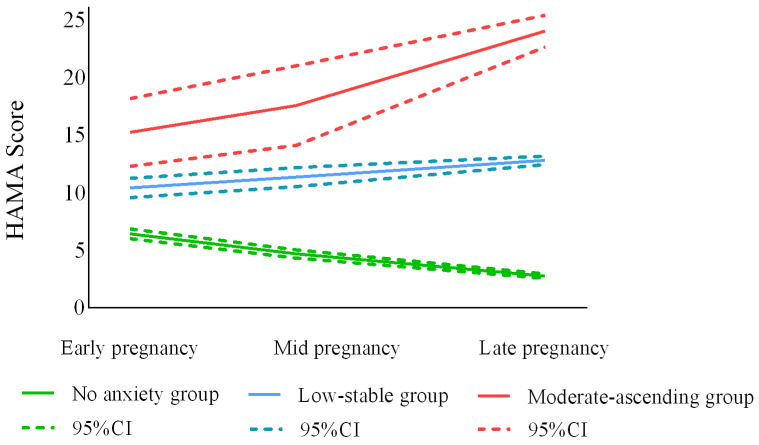
Optimal model trajectory curves for prenatal anxiety (n = 916).

Of the three anxiety trajectory groups (Figure 2), the “no significant anxiety” (n = 669, 73.0%) group was the largest, with more than 90% of the women reporting no anxiety symptoms (Table 3). The “low-stable” group (n = 216, 23.6%) consisted of women who scored between the other two groups, some of whom had suspected anxiety symptoms. Women in the “moderate-ascending” trajectory group (n = 31, 3.4%) were prone to increased anxiety symptoms during pregnancy, and they suffered from mild anxiety in early and middle pregnancy and moderate anxiety in late pregnancy.

#### 3.3.3. Trajectory Groups of Prenatal Depression Symptoms

**Figure 3:**
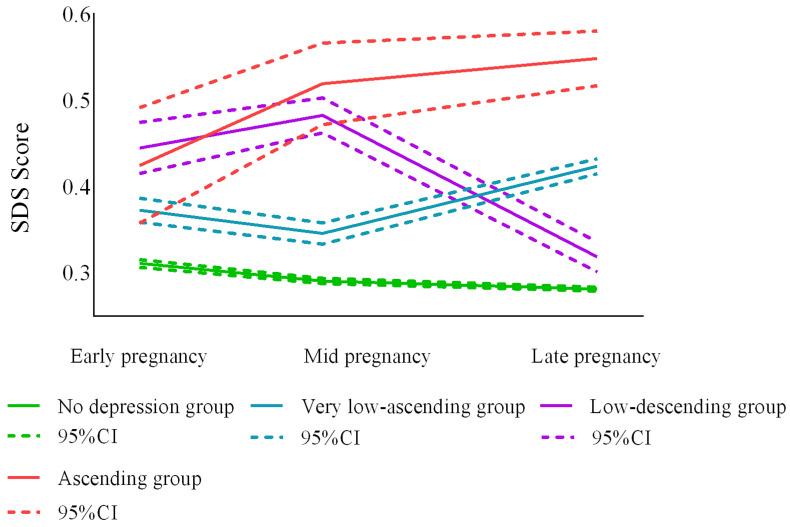
Optimal model trajectory curves for prenatal depression (n = 916).

Among the four depression trajectory groups (Figure 3), the “no depression” group (n = 749, 81.8%) and the “very low-ascending” group (n = 99, 10.8%) had consistently low depression scores during follow-up, but the “very low” group had higher scores than the “no depression” group. The “low-descending” group (n = 50, 5.5%) had the highest scores in early pregnancy, and women in this group had increased anxiety symptoms in middle pregnancy. the “low-ascending” group (n = 18, 2.0%) included women with relatively high baseline levels who were prone to mild depressive symptoms in middle and late pregnancy (Table 3).

### 3.4. Predictors of Trajectory Subgroup Membership

Univariate analysis was performed based on the 18 demographic and psychosocial factors (Table 4). Factors with *p* < 0.1 in the chi-squared analysis were entered into a multinomial logistic regression to examine the association between different trajectories and personal, family, and social factors.

#### 3.4.1. Stress Trajectory Groups

The low-stable group was used as a reference. Women who had completed college, a bachelor’s degree, or further higher education; attended antenatal classes; or reported vaginal bleeding in early pregnancy were at higher risk of “early pregnancy stress” (OR = 7.61, 95% CI = 1.74–33.27; OR = 0.30, 95% CI = 0.11–0.79; OR = 5.35, 95% CI = 1.55–18.45). Living in less developed regions, low levels of family care, and social support were risk factors for “moderate-ascending stress” (OR = 2.47, 95% CI = 1.66–3.68; OR = 1.95, 95% CI = 1.29–2.96; OR = 2.39, 95% CI = 1.56–3.67). The results are presented in Table 5.

#### 3.4.2. Anxiety Trajectory Groups

The no-anxiety group was set as the reference. Living in a rural area, having taken teratogenic drugs, owning pets, and low levels of family care or social support were significantly associated with the ”moderate-ascending“ group (OR = 0.37, 95% CI = 0.17–0.79; OR = 3.85, 95% CI = 1.11–13.34; OR = 4.10, 95% CI = 1.21–13.84; OR = 2.42, 95% CI = 1.09–5.38; OR = 3.24, 95% CI = 1.35–7.76). Women with these characteristics were at higher risk of experiencing the development and persistence of moderate or severe anxiety symptoms during pregnancy. The results are presented in Table 6.

#### 3.4.3. Depression Trajectory Groups

The “no depression” trajectory group was used as a reference. Insufficient family care (OR = 3.07, 95% CI = 1.08–8.71) and poor social support (OR = 3.54, 95% CI = 1.09–11.48) significantly increased the risk of depression in pregnant women (Table 7).

## 4. Discussion

Currently, most studies on prenatal stress, anxiety, and depression are cross-sectional and tend to focus on the overall picture, ignoring the heterogeneity in individual development. This study showed that, although the overall prevalence of stress, anxiety, and depressive symptoms tended to decrease, most pregnant women in the moderate-ascending stress group, moderate-ascending anxiety group, and low-ascending depression group showed increases in symptoms with increasing gestational age, and some women did not experience stress, anxiety, or depressive symptoms until middle or late pregnancy. Therefore, it was necessary to group the trajectories of stress, anxiety, and depression in pregnant women using a trajectory analysis model. This longitudinal study identified different trajectory groups of stress, anxiety, and depression symptoms during pregnancy among women in southwest China and explored the factors that potentially influenced them.

### 4.1. Profile of Trajectory Group Membership

Depression and anxiety symptoms are common negative psychological emotions in pregnant women, but only a limited number of studies have investigated anxiety and depression trajectory groups. We identified three anxiety trajectory groups, in agreement with Ahmed et al. [32]. However, some studies have presented different results, such as that by Shu-Yu et al. [33], who measured anxiety and depressive symptoms in 139 Taiwanese women who underwent elective cesarean delivery in late pregnancy and at 1 day postpartum, 1 week postpartum, and 6 months postpartum, ultimately identifying four anxiety trajectory groups. Furthermore, Bayrampour H et al. [11] recruited 1445 Canadian women and measured their anxiety and depression symptoms in middle and late pregnancy and at 4 and 12 months postpartum, identifying five anxiety trajectory groups. The study designs in these two studies differed significantly from ours, particularly in terms of the time points assessed and the numbers and characteristics of participants, which may be an important reason for the inconsistent numbers of trajectories. In addition, we concluded that more than 70% of women did not experience or were suspected to experience anxiety symptoms during pregnancy and about 27% may have experienced significant anxiety symptoms, corresponding to the 24% prevalence of prenatal anxiety reported by others [11,32,33]. Of concern is the “moderate-ascending” (3.4%) group; it was the smallest trajectory group, but women in this group experienced persistent increases in anxiety symptoms during pregnancy, making it a high-risk group for anxiety symptoms.

We identified four depression trajectory groups. The depression trajectory showed more pronounced fluctuations and more detailed groupings compared to anxiety, which was consistent with Ahmed’s study [32]. Xu et al. [35] studied the trajectory groups of perinatal depression in women in Hunan Province, China, and found three trajectory groups, reporting the same trends in the antenatal period as the three largest trajectory groups in the present study. Xu et al. [35] found that 90% of women in the perinatal period were stable and free of depression, and Mora [43] and Sutter-Dallay [44] found that more than 75% of women did not experience perinatal depressive symptoms; these findings are close to the results of the present study (81.8%). However, Ahmed et al. [32] and Bayrampour et al. [11] reported moderate or mild depressive symptoms in about 40% of their respective trajectory groups, and the high detection rates may have been related to the use of different depression screening tools, with both studies using the EPDS to assess depressive symptoms. They may also have been related to sample characteristics (income, ethnicity, and region), in addition to the fact that the sample in this study consisted of low-risk women without health problems, such as mental illness, which may have contributed to the low prevalence of depressive symptoms.

A few studies have focused on the stress trajectory group, as some studies have identified stress as an influential factor and suggested that stress levels are associated with the anxiety and depression symptom trajectory groups [45]. However, it has also been suggested that stress during pregnancy is associated with negative emotions during pregnancy and childbirth and that a causal relationship between stress and symptoms of depression and anxiety cannot be established even by longitudinal analyses [34]. We found a high prevalence of stress in Chinese women [7,46,47], and to further understand the trajectory of stress during pregnancy in Chinese women, we conducted an independent trajectory analysis of stress symptoms in this study. We identified three stress trajectory groups, two of which contained distinct characteristics: the group with early pregnancy stress and the group with persistently elevated stress during pregnancy. Women with early pregnancy stress could reduce their stress symptoms through self-regulation, whereas women in the persistently elevated stress symptom trajectory group required more of our attention. Approximately 20% of women experience moderate or severe stress symptoms during pregnancy.

### 4.2. Risk Factors for Trajectory Subgroups

Family care was found to be one of the important influencing factors in the antenatal stress, anxiety, and depression trajectory groups, which was consistent with previous studies [11,48]. The birth of a child triggers a woman’s need for family companionship and care, and a positive family environment not only enhances a woman’s confidence in giving birth but also promotes the health of the mother and fetus [46]. Conversely, inadequate support and care from the family put pregnant women at higher risk of stress, anxiety, and depressive symptoms. The level of social support is also a significant predictor of stress, anxiety, and depressive symptom trajectory groups. Social support includes support from family, friends, neighbors, and co-workers [49], as well as help from social groups in terms of informational, material, and emotional support [50]. Social support is an effective factor in women’s responses to pregnancy-related problems, and good communication with friends, relatives, and neighbors can provide pregnant women with sufficient parenting experiences to prepare them for motherhood [48]. In addition, adequate social support helps women of childbearing age gain knowledge, improve their self-confidence, and reduce their stress and burdens [51,52]. All these kinds of support are beneficial in alleviating the negative emotions of pregnant women.

We also found that education level was associated with the stress trajectory groups [53,54,55] and that women with higher levels of education were more likely to have high levels of stress symptoms in early pregnancy, which may stem from heavy workload or the pressure to exceed expectations for the next generation. Women with vaginal bleeding were at higher risk of experiencing stress symptoms in the first trimester, but as the weeks of pregnancy progressed and the weeks with a high propensity for miscarriage passed without incident, stress symptoms gradually decreased and returned to normal in late pregnancy. In addition, women in less economically developed areas were more likely to experience high and consistently increasing levels of stress during pregnancy. The reasons behind this are complex and may be related to economic conditions, with some studies finding poverty or low income to be risk factors for prenatal stress in pregnancy [23,56]; however, there was no direct association between household per capita income and stress trajectory groups in this study. Less developed areas are often far from city centers and have relatively poor medical and educational resources, which is not conducive to the education and growth of children.

Women who had taken potentially teratogenic medications or owned pets were more likely to experience persistent and worsening anxiety symptoms during pregnancy due to concerns about the side effects of medications or parasitic infections. In addition, women living in rural areas were also at higher risk of experiencing persistent worsening of anxiety symptoms during pregnancy than women living in urban areas. This may be related to the economic level and living conditions, with relatively poor healthcare being an additional driver of prenatal anxiety symptoms [57,58]. In addition, lack of knowledge about maternal health and poor access to health services may lead to greater anxiety among rural women.

This study is one of the few longitudinal studies in China that repeatedly measured psychological changes during pregnancy, and it is of great significance. However, the study only included the gestational period without the postpartum period and lacked other factors, such as the previous history of anxiety/depression, marital status, etc. In addition, the criteria for the recruitment of volunteers in this study excluded women with a history of cesarean delivery or a history of mental illness, which may have led to biased results. Therefore, studies with larger sample sizes and more factors are recommended to determine the most predictive characteristics.

## 5. Limitations

This study had several limitations. The participants were from only one province in China. In addition, due to the exclusion criteria, none of the women had a history of cesarean delivery. Therefore, the results of this study are not representative of all pregnant women in mainland China.

## 6. Conclusions

This study found that maternal stress, anxiety, and depressive symptoms exhibited several different trajectory groups and were all associated with family care and social support. In contrast to anxiety symptoms, both stress and depressive symptoms fluctuated significantly after early pregnancy assessment. Therefore, regular psychological screening of high-risk women is necessary, and early screening for mental health is important to assist healthcare providers in identifying women at risk of adverse pregnancy outcomes.

In addition, national health departments should pay more attention to the mental health of pregnant women in less developed and rural areas, and hospitals could provide more free counseling and assistance in rural areas. Family care and social support also have a great impact on the mental health of pregnant women, and it is recommended that healthcare providers do more among the families of pregnant women and in relation to the societies they live in, such as encouraging family members to participate in prenatal health education for pregnant women and providing a platform for family members to consult regarding perinatal care. Society and public media should also advocate for more care and understanding for pregnant women and provide a better socio-humanistic environment for women that is conducive to pregnancy. These measures may contribute to women’s prenatal mental health and have important implications for maternal and child health.

## Figures and Tables

**Table 1:** Prevalence of stress, anxiety and depressive symptoms (n (%)).

Trimester	Stress	Anxiety	Depression
Early pregnancy			
No	71 (7.75)	787 (85.92)	872 (95.20)
Mild	703 (76.75)	99 (10.81)	40 (4.37)
Moderate	136 (14.85)	25 (2.73)	2 (0.22)
Severe	6 (0.66)	5 (0.55)	2 (0.22)
Middle pregnancy			
No	88 (9.61)	813 (88.76)	882 (96.29)
Mild	717 (78.28)	69 (7.53)	26 (2.84)
Moderate	108 (11.79)	30 (3.28)	7 (0.76)
Severe	3 (0.33)	4 (0.44)	1 (0.11)
Late pregnancy			
No	104 (11.35)	824 (89.96)	891 (97.27)
Mild	715 (78.06)	73 (7.97)	22 (2.40)
Moderate	96 (10.48)	18 (1.97)	3 (0.33)
Severe	1 (0.11)	1 (0.11)	0 (0)

**Table 2:** Mean scores and confidence intervals for trajectory groups, and comparison of the subgroups of stress, anxiety, and depression at each follow-up point.

Variable	Subgroup	Trimester		
		Early Pregnancy	Middle Pregnancy	Late Pregnancy
Stress	Low-stable	0.44 (0.42–0.46) ^a^	0.39 (0.36–0.41) ^a^	0.33 (0.31–0.35)^a^
	High-descending	1.69 (1.55–1.83) ^b^	1.17 (0.87–1.47) ^b^	0.23 (0.12–0.33) ^b^
	Moderate-ascending	1.00 (0.93–1.07) ^c^	1.03 (0.96–1.10) ^c^	1.22 (1.17–1.27) ^c^
Anxiety	No significant anxiety	6.41 (5.99–6.83) ^a^	4.67 (4.31–5.02) ^a^	2.74 (2.55–2.93) ^a^
	Low-stable	10.38 (9.54–11.22) ^b^	11.32 (10.49–12.15) ^b^	12.77 (12.40–13.13) ^b^
	Moderate-ascending	15.19 (12.25–18.13) ^c^	17.52 (14.07–20.96) ^c^	23.97 (22.59–25.34) ^c^
Depression	No depression	0.311 (0.306–0.315) ^a^	0.291 (0.288–0.294) ^a^	0.281 (0.279–0.284) ^a^
	Very low-ascending	0.373 (0.359–0.387) ^b^	0.346 (0.333–0.358) ^b^	0.423 (0.415–0.432) ^b^
	Low-descending	0.445 (0.415–0.474) ^c^	0.482 (0.462–0.502) ^c^	0.319 (0.301–0.336) ^c^
	Low-ascending	0.424 (0.358–0.491) ^b,c^	0.519 (0.472–0.566) ^c^	0.548 (0.517–0.580) ^d^

Note: ^a–d^ No statistical difference between groups with the same letter at the time point.

**Table 3:** Distributions of symptom levels in the stress, anxiety, and depression trajectory subgroups at each follow-up point.

Degree	Stress Trajectory Groups, n (%)			Anxiety Trajectory Groups, n (%)			Depression Trajectory Groups, n (%)			
	Low-Stable	High-Descending	Moderate-Ascending	No Significant Anxiety	Low-Stable	Moderate-Ascending	No Depression	Very Low-Ascending	Low-Descending	Low-Ascending
Early pregnancy										
None	70 (9.3)	0 (0)	1 (0.7)	611 (91.3)	162 (75.0)	14 (45.2)	733 (97.9)	91 (91.9)	35 (70.0)	13 (72.2)
Mild	629 (83.9)	0 (0)	74 (52.1)	46 (6.9)	42 (19.4)	11 (35.5)	15 (2.0)	8 (8.1)	13 (26.0)	4 (22.2)
Moderate	51 (6.8)	20 (83.3)	65 (45.8)	10 (1.5)	11 (5.1)	4 (12.9)	1 (0.1)	0 (0)	1 (2.0)	0 (0)
Severe	0 (0)	4 (16.7)	2 (1.4)	2 (0.3)	1 (0.5)	2 (6.4)	0 (0)	0 (0)	1 (2.0)	1 (5.6)
Middle pregnancy										
None	85 (11.3)	2 (8.3)	1 (0.7)	640 (95.7)	163 (75.4)	10 (32.3)	749 (100)	99 (100)	27 (54.0)	7 (38.9)
Mild	638 (85.1)	6 (25.0)	73 (51.4)	22 (3.3)	38 (17.6)	9 (29.0)	0 (0)	0 (0)	17 (34.0)	9 (50.0)
Moderate	27 (3.6)	14 (58.3)	67 (47.2)	7 (1.0)	12 (5.6)	11 (35.5)	0 (0)	0 (0)	6 (12.0)	1 (5.6)
Severe	0 (0)	2 (8.3)	1 (0.7)	0 (0)	3 (1.4)	1 (3.2)	0 (0)	0 (0)	0 (0)	1 (5.6)
Late pregnancy										
None	98 (13.1)	6 (25.0)	0 (0)	669 (100)	155 (71.8)	0 (0)	749 (100)	88 (88.9)	50 (100)	4 (22.2)
Mild	650 (86.7)	18 (75.0)	47 (33.1)	0 (0)	61 (28.2)	12 (38.7)	0 (0)	11 (11.1)	0 (0)	11 (61.1)
Moderate	2 (0.2)	0 (0)	94 (66.2)	0 (0)	0 (0)	18 (58.1)	0 (0)	0 (0)	0 (0)	3 (16.7)
Severe	0 (0)	0 (0)	1 (0.7)	0 (0)	0 (0)	1 (3.2)	0 (0)	0 (0)	0 (0)	0 (0)

**Table 4:** Characteristics of and correlations for stress, anxiety, and depression trajectory subpopulations.

Characteristics	Total (n = 916)	Latent Stress Trajectory Groups (n%)			*p*-Value	Latent Anxiety Trajectory Groups (n%)			*p*-Value	Latent Depression Trajectory Groups (n%)				*p*-Value
		Low Risk	High Risk	Early Stage		Low Risk	Mild Anxiety	High Risk		Very Low Risk	Low Risk	Middle Stage	High Risk	
Age					0.0222				0.3725					0.5995
<25 years	396 (43.2)	309 (41.2)	72 (50.7)	15 (62.5)		280 (41.9)	100 (46.3)	16 (51.6)		317 (42.3)	46 (46.5)	23 (46.0)	10 (55.6)	
≥25 years	508 (55.5)	430 (57.3)	69 (48.6)	9 (37.5)		379 (56.7)	114 (52.8)	15 (48.4)		422 (56.3)	51 (51.5)	27 (54.0)	8 (44.4)	
Missing	12 (1.3)	11 (1.5)	1 (0.7)	0 (0)		10 (1.5)	2 (0.9)	0 (0)		10 (1.3)	2 (2.0)	0 (0)	0 (0)	
Region					<0.0001				<0.0001					0.005
Developed	355 (38.8)	264 (35.2)	82 (57.7)	9 (37.5)		227 (33.9)	112 (51.9)	16 (51.6)		271 (36.2)	47 (47.5)	26 (52.0)	11 (61.1)	
Less developed	561 (61.2)	486 (64.8)	60 (42.3)	15 (62.5)		442 (66.1)	104 (48.1)	15 (48.4)		478 (63.8)	52 (52.5)	24 (48.0)	7 (38.9)	
Residence					0.7057				0.0148					0.6501
Urban	570 (62.2)	469 (62.5)	88 (62.0)	13 (54.2)		416 (62.2)	142 (65.7)	12 (38.7)		472 (63.0)	57 (57.6)	29 (58.0)	12 (66.7)	
Rural	346 (37.8)	281 (37.5)	54 (38.0)	11 (45.8)		253 (37.8)	74 (34.3)	19 (61.3)		277 (37.0)	42 (42.4)	21 (42.0)	6 (33.3)	
Educational level					0.0085				0.2628					0.5276
High school or below	555 (60.6)	457 (60.9)	76 (53.5)	22 (91.7)		396 (59.2)	137 (63.4)	22 (71.0)		448 (59.8)	63 (63.6)	34 (68.0)	10 (55.6)	
College or above	361 (39.4)	293 (39.1)	66 (46.5)	2 (8.3)		273 (40.8)	79 (36.6)	9 (29.0)		301 (40.2)	36 (36.4)	16 (32.0)	8 (44.4)	
Occupational status					0.0851				0.0477					0.0948
Employed	451 (49.2)	381 (50.8)	62 (43.7)	8 (33.3)		340 (50.8)	102 (47.2)	9 (29.0)		383 (51.1)	41 (41.4)	21 (42.0)	6 (33.3)	
Housewife/unemployed	465 (50.8)	369 (49.2)	80 (56.3)	16 (66.7)		329 (49.2)	114 (52.8)	22 (71.0)		366 (48.9)	58 (58.6)	29 (58.0)	12 (66.7)	
Monthly per capita household income (CNY)					0.3067				0.3876					0.2729
≤3000	232 (25.3)	192 (25.6)	35 (24.6)	5 (20.8)		175 (26.2)	47 (21.8)	10 (32.3)		191 (25.5)	18 (18.2)	17 (34.0)	6 (33.3)	
3000–5000	380 (41.5)	304 (40.5)	68 (47.9)	8 (33.3)		267 (39.9)	100 (46.3)	13 (41.9)		304 (40.6)	46 (46.5)	22 (44.0)	8 (44.4)	
≥5001	303 (33.1)	253 (33.8)	39 (27.5)	11 (45.8)		226 (33.8)	69 (31.9)	8 (25.8)		253 (33.8)	35 (35.4)	11 (22.0)	4 (22.2)	
Missing	1 (0.1)	1 (0.1)	0 (0)	0 (0)		1 (0.1)	0 (0)	0 (0)		1 (0.1)	0 (0)	0 (0)	0 (0)	
Prenatal exercise					0.0096				0.0252					0.6773
Regular	584 (63.8)	495 (66.0)	75 (52.8)	14 (58.3)		443 (66.2)	121 (56.0)	20 (64.5)		479 (64.0)	64 (64.6)	32 (64.0)	9 (50)	
Never or occasional	332 (35.2)	255 (34.0)	67 (47.2)	10 (41.7)		226 (33.8)	95 (44.0)	11 (35.5)		270 (36.0)	35 (35.4)	18 (36.0)	9 (50)	
Antenatal classes					0.0406				0.0894					0.8053
Yes	455 (49.7)	369 (49.2)	68 (47.9)	18 (75.0)		343 (51.3)	94 (43.5)	18 (58.1)		368 (49.1)	53 (53.5)	26 (52.0)	8 (44.4)	
No	461 (50.3)	381 (50.8)	74 (52.1)	6 (25.0)		326 (48.7)	122 (56.5)	13 (41.9)		381 (50.9)	46 (46.5)	24 (48.0)	10 (55.6)	
Parity					0.0226				0.7730					0.5685
0 (primiparity)	618 (67.5)	491 (65.5)	109 (76.8)	18 (75.0)		447 (66.8)	150 (69.4)	21 (67.7)		500 (66.8)	67 (67.7)	38 (76.0)	13 (72.2)	
≥1	298 (32.5)	259 (34.5)	33 (23.2)	6 (25.0)		222 (33.2)	66 (30.6)	10 (32.3)		249 (33.2)	32 (32.3)	12 (24.0)	5 (27.8)	
Vaginal bleeding during early pregnancy					0.0208				0.3186					0.0389
Yes	51 (5.6)	36 (4.8)	11 (7.7)	4 (16.7)		33 (4.9)	15 (6.9)	3 (9.7)		36 (4.8)	12 (12.1)	2 (4.0)	1 (5.6)	
No	865 (94.4)	714 (95.2)	131 (92.3)	20 (83.3)		636 (95.1)	201 (93.1)	28 (90.3)		713 (95.2)	87 (87.9)	48 (96.0)	17 (94.4)	
Use of possible teratogenic drugs					0.5059				0.0007					0.4935
Yes	41 (4.5)	31 (4.1)	9 (6.3)	1 (4.2)		20 (3.0)	17 (7.9)	4 (12.9)		31 (4.1)	5 (5.1)	3 (6.0)	2 (11.1)	
No	875 (95.5)	719 (95.9)	133 (93.7)	23 (95.8)		649 (97.0)	199 (92.1)	27 (87.1)		718 (95.9)	94 (94.9)	47 (94.0)	16 (88.9)	
Exposure to high concentrations of industrial chemicals					0.6329				0.0181					0.0586
Yes	5 (0.5)	4 (0.5)	1 (0.7)	0 (0)		1 (0.1)	3 (1.4)	1 (3.2)		2 (0.3)	2 (2.0)	1 (2.0)	0 (0)	
No	911 (99.5)	746 (99.5)	141 (99.3)	24 (100)		668 (99.9)	213 (98.6)	30 (96.8)		747 (99.7)	97 (98.0)	49 (98.0)	18 (100)	
Ownership of pets					0.0905				0.0023					0.0001
Yes	35 (3.8)	24 (3.2)	10 (7.0)	1 (4.2)		18 (2.7)	13 (6.0)	4 (12.9)		18 (2.4)	10 (10.1)	5 (10.0)	2 (11.1)	
No	881 (96.2)	726 (96.8)	132 (93.0)	23 (95.8)		651 (97.3)	203 (94.0)	27 (87.1)		731 (97.6)	89 (89.9)	45 (90.0)	16 (88.9)	
Exposure to newly renovated houses					0.0037				0.1623					0.6433
Yes	9 (1.0)	4 (0.5)	5 (3.5)	0 (0)		5 (0.7)	3 (1.4)	1 (3.2)		7 (0.9)	1 (1.0)	1 (2.0)	0 (0)	
No	907 (99.0)	746 (99.5)	137 (96.5)	24 (100)		664 (99.3)	213 (98.6)	30 (96.8)		742 (99.1)	98 (99.0)	49 (98.0)	18 (100)	
Drinking history					0.0592				0.0734					0.0035
Yes	11 (1.2)	6 (0.8)	5 (3.5)	0 (0)		5 (0.7)	5 (2.3)	1 (3.2)		5 (0.7)	3 (3.0)	1 (2.0)	2 (11.1)	
No	905 (98.8)	744 (99.2)	137 (96.5)	24 (100)		664 (99.3)	211 (97.7)	30 (96.8)		744 (99.3)	96 (97.0)	49 (98.0)	16 (88.9)	
Smoking history					0.0960				0.1480					0.0094
Active/passive smoking	27 (3.0)	19 (2.5)	6 (4.2)	2 (8.3)		16 (2.4)	9 (4.2)	2 (6.5)		17 (2.3)	6 (6.1)	2 (4.0)	2 (11.1)	
No smoking	889 (97.0)	731 (97.5)	136 (95.8)	22 (91.7)		653 (97.6)	207 (95.8)	29 (93.5)		732 (97.7)	93 (93.9)	48 (96.0)	16 (88.9)	
Previous medical history					0.7418				0.0105					0.1544
Yes	68 (7.4)	55 (7.3)	12 (8.5)	1 (4.2)		41 (6.1)	26 (12.0)	1 (3.2)		51 (6.8)	12 (12.1)	5 (10.0)	0 (0)	
No	848 (92.6)	695 (92.7)	130 (91.5)	23 (95.8)		628 (93.9)	190 (88.0)	30 (96.8)		698 (93.2)	87 (87.9)	45 (90.0)	18 (100)	
Level of family care					<0.0001				<0.0001					<0.0001
Low	317 (34.6)	234 (31.2)	77 (54.2)	6 (25.0)		195 (29.1)	103 (47.7)	19 (61.3)		226 (30.2)	51 (51.5)	28 (56.0)	12 (66.7)	
High	599 (65.4)	516 (68.8)	65 (45.8)	18 (75.0)		474 (70.9)	113 (52.3)	12 (38.7)		523 (69.8)	48 (48.5)	22 (44.0)	6 (33.3)	
Level of social support					<0.0001				<0.0001					<0.0001
Low	414 (45.2)	309 (41.2)	93 (65.5)	12 (50.0)		267 (39.9)	124 (57.4)	23 (74.2)		303 (40.5)	60 (60.6)	37 (74.0)	14 (77.8)	
High	502 (54.8)	441 (58.8)	49 (34.5)	12 (50.0)		402 (60.1)	92 (42.6)	8 (25.8)		446 (59.5)	39 (39.4)	13 (26.0)	4 (22.2)	

**Table 5:** Multinomial logistic regression analysis of stress trajectory groups.

Variables	High-Descending			Moderate-Ascending		
	*p*-Value	OR	95% CI	*p*-Value	OR	95% CI
Region: developed = 1; less developed = 2	0.710	1.20	0.47–3.08	<0.0001	2.47	1.66–3.68
Education level: high school degree or below = 1; bachelor’s degree or above = 2	0.007	7.61	1.74–33.27	0.037	0.65	0.44–0.97
Antenatal classes: yes = 1; no = 2	0.015	0.30	0.11–0.79	0.209	0.78	0.52–1.15
Vaginal bleeding: no = 1; yes = 2	0.008	5.35	1.55–18.45	0.594	1.23	0.58–2.61
Level of family care: high = 1; low = 2	0.175	0.50	0.18–1.37	0.002	1.95	1.29–2.96
Level of social support: high = 1; low = 2	0.315	1.59	0.65–3.91	<0.0001	2.39	1.56–3.67

**Table 6:** Multinomial logistic regression analysis of anxiety trajectory groups.

Variables	Low-Stable			Moderate-Ascending		
	*p*-Value	OR	95% CI	*p*-Value	OR	95% CI
Region: developed = 1; less developed = 2	<0.0001	2.00	1.43–2.81	0.143	1.80	0.819–3.96
Residence: rural = 1; town = 2	0.477	1.13	0.80–1.59	0.010	0.37	0.17–0.79
Use of teratogenic drugs: no = 1; yes = 2	0.113	1.81	0.87–3.79	0.033	3.85	1.11–13.34
Owning pets: no = 1; yes = 2	0.138	1.79	0.83–3.89	0.023	4.10	1.21–13.84
Level of family care: high = 1; low = 2	0.002	1.76	1.24–2.51	0.029	2.42	1.09–5.38
Level of social support: high = 1; low = 2	0.005	1.65	1.16–2.33	0.008	3.24	1.35–7.76

**Table 7:** Multinomial logistic regression analysis of depression trajectory groups.

Variables	Very Low-Ascending			Low-Descending			Low-Ascending		
	*p*-Value	OR	95% CI	*p*-Value	OR	95% CI	*p*-Value	OR	95% CI
Owning pets: no = 1; yes = 2	0.001	4.29	1.88–9.79	0.006	4.52	1.54–13.21	0.068	4.34	0.90–20.94
Level of family care: high = 1; low = 2	0.003	2.05	1.28–3.26	0.070	1.83	0.95–3.51	0.035	3.07	1.08–8.71
Level of social support: high = 1; low = 2	0.016	1.79	1.12–2.87	0.001	3.62	1.74–7.54	0.035	3.54	1.09–11.48

## Data Availability

All data included in the current study can be obtained from the corresponding author through their email address upon reasonable request.

## Appendix A

**Table A1:** GMM fitting statistics results (prenatal stress).

Subgroup Number/Parameter	AIC	BIC	aBIC	Entropy	VLRT (*p*-Value)	Composition Ratio
Linear						
1	2351.054	2389.614	2364.207	-	-	1
2	2143.180	2196.200	2161.265	0.856	<0.0001	0.840/0.160
3	2036.929	2104.410	2059.947	0.892	0.0038	0.026/0.155/0.819
4	1956.901	2038.841	1984.851	0.868	0.0521	0.022/0.224/0.692/0.062
Quadratic						
1	2774.138	2807.878	2785.647	-	-	1
2	2147.336	2224.456	2173.642	0.856	0.2398	0.840/0.160
3	2004.625	2101.025	2037.507	0.873	0.2398	0.060/0.157/0.783
4	1901.191	2016.872	1940.651	0.899	0.2398	0.782/0.052/0.015/0.151
Free						
1	2349.010	2392.390	2363.807	-	-	1
2	1611.409	1688.529	1637.715	0.618	<0.0001	0.469/0.531
3	1571.809	1682.670	1609.625	0.711	0.0101	0.338/0.533/0.129
4	1489.272	1633.872	1538.596.	0.755	0.5005	0/0.100/0.403/0.497

Note: “-” means no value.

**Table A2:** GMM fitting statistics results (prenatal anxiety).

Subgroup Number/Parameter	AIC	BIC	aBIC	Entropy	VLRT (*p*-Value)	Composition Ratio
Linear						
1	17,110.001	17,148.561	17,123.154	-	-	1
2	16,900.303	16,953.323	16,918.388	0.845	0.0026	0.805/0.196
3	16,754.626	16,822.106	16,777.644	0.903	0.0004	0.236/0.034/0.730
4	16,661.442	16,743.383	16,689.393	0.889	0.0052	0.025/0.236/0.706/0.032
Quadratic						
1	17,435.509	17,469.249	17,447.018	-	-	1
2	16,902.059	16,979.179	16,928.365	0.849	0.2397	0.138/0.862
3	16,776.928	16,873.328	16,809.811	0.880	0.2398	0.032/0.182/0.786
4	16,617.536	16,733.217	16,656.996	0.904	0.2397	0.141/0.770/0.046/0.044
Free						
1	17,106.952	17,150.332	17,121.749	-	-	1
2	16,262.719	16,339.839	16,289.025	0.776	<0.0001	0.602/0.398
3	16,062.945	16,173.806	16,100.761	0.740	<0.0001	0.416/0.374/0.210
4	16,037.170	16,162.491	16,079.918	0.792	0.0011	0.366/0.414/0.209/0.012

Note: “-” means no value.

**Table A3:** GMM fitting statistics results (prenatal depression).

Subgroup Number/Parameter	AIC	BIC	aBIC	Entropy	VLRT	Composition Ratio
Linear						
1	−7203.861	−7165.300	−7190.707	-	-	1
2	−7547.183	−7494.163	−7529.098	0.930	0.0692	0.890/0.110
3	−7675.506	−7608.025	−7652.487	0.915	0.0088	0.040/0.127/0.833
4	−7750.502	−7668.561	−7722.551	0.904	0.1147	0.109/0.785/0.096/0.010
Quadratic						
1	−6993.328	−6959.588	−6981.819	-	-	1
2	−7553.662	−7476.542	−7527.356	0.933	0.2398	0.897/0.103
3	−7680.756	−7608.456	−7656.094	0.934	0.0052	0.871/0.044/0.085
4	−7800.462	−7708.881	−7769.223	0.940	<0.001	0.818/0.108/0.055/0.019
Free						
1	−7215.817	−7172.491	7201.073	-	-	1
2	−8296.742	−8219.622	−8270.436	0.736	<0.0001	0.390/0.610
3	-	-	-	-	-	-
4	-	-	-	-	-	-

Note: “-” means no value.
